# Paraprobiotics and Postbiotics—Current State of Scientific Research and Future Trends toward the Development of Functional Foods

**DOI:** 10.3390/foods12122394

**Published:** 2023-06-16

**Authors:** Shênia Santos Monteiro, Carlos Eduardo Schnorr, Matheus Augusto de Bittencourt Pasquali

**Affiliations:** 1 Graduate Program in Engineering and Natural Resource Management, Center for Technology and Natural Resources, Federal University of Campina Grande, Campina Grande CEP 58429-140, Paraíba, Brazil; 2 Departamento de Ciencias Naturales y Exactas, Universidad de la Costa, CUC, Calle 58 55–66, Barranquilla 080002, Atlántico, Colombia; 3 Department of Food Engineering, Federal University of Campina Grande, Campina Grande CEP 58429-140, Paraíba, Brazil

**Keywords:** probiotics, bibliometric analysis, bioactivity, food safety

## Abstract

The potential of paraprobiotics and postbiotics to be used as beneficial agents for human health has caused an effort by the scientific community to gather information about the bioactivity of these compounds and production methods. Understanding the evolution of scientific research in this area of study is important to understand the future perspectives and the main bottlenecks of scientific and technological development involving these compounds. In this scenario, this review work used a bibliometric analysis tool intending to improve the scientific documentation, bringing information and communicating the results to the scientific community through the quantitative analysis of the current literature, available in one of the main databases, the Web of Science, also providing recent information on the evolution and future perspectives in the field of paraprobiotic and postbiotic development. The results of this study showed that the main studies discuss the bioactivity of these compounds. Concerning the development of functional foods, there is a need for extensive research on production methods and the interaction of these compounds with food. However, it concluded that much still needs to be studied to prove the claims of bioactivity, especially when used for the development of functional foods.

## 1. Introduction

Food is currently not only seen as a means of satisfying hunger but has become an important mediator in maintaining the health and well-being of the world’s population [1]. This has changed the pattern of food development. In recent years, the focus has been on developing new food products, emphasizing the use of probiotics, paraprobiotics, and postbiotic microorganisms [2].

**Figure 1:**
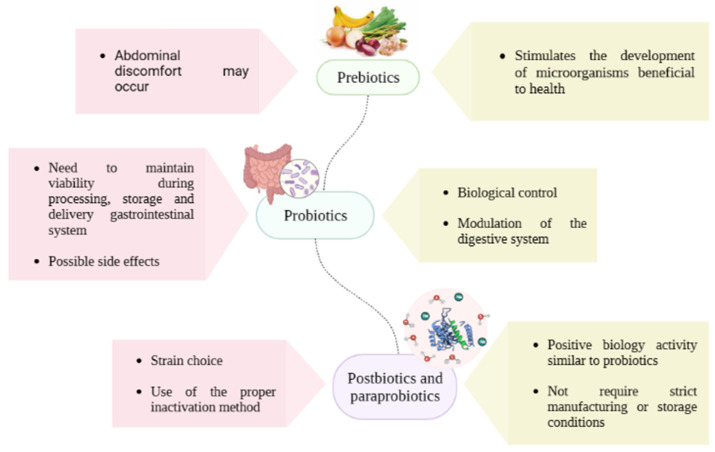
Positive highlights and challenges related to prebiotics, probiotics, probiotics, and paraprobiotics.

Some species of lactic acid bacteria are recognized as having specific health effects and are called probiotics [3]. Probiotics are live microorganisms that, when administered in adequate amounts, confer a health benefit on the host [4]. However, emerging concerns arise about safety issues associated with the administration of probiotic bacteria [5]. To deal with such issues and limitations of probiotics, such as unknown molecular mechanisms, specific retention behavior, short duration of action, development of antibiotic resistance, ambiguous beneficial effects, maintenance of viability and stability in the production process, the paraprobiotics and postbiotic derivatives of probiotics are favorable alternatives for the development of new products with benefits for human health and well-being [6]. A summary of the main strengths and weaknesses attributed to prebiotics, probiotics, postbiotics, and paraprobiotics is shown in Figure 1.

Paraprobiotics are inactivated microbial intact (nonviable) cells, previously referred to in the literature as “inactivated probiotics” and phantom probiotics”, or cell fractions, which have completely lost their viability after exposure to factors that alter microbial cell structures, such as breakage strand DNA (Deoxyribonucleic acid), disruption of the cell membrane, or mechanical damage to the cell envelope, but which conferred benefits on the consumer’s health [7]. Postbiotics, on the other hand, are soluble factors, such as metabolic products or by-products secreted by live bacteria or released after bacterial lysis, that can offer a positive effect on the consumer [8]. These components have production advantages over probiotics since cell viability is not required. Therefore, it makes technological development and application in food more favorable.

Given the growing interest in the development of products that are beneficial to health and safety, it is important to clarify the current situation of the literature on paraprobiotics and postbiotics to understand the path of the industry in the development of functional foods, which seeks to meet the demand of the modern consumer. Bibliometric analysis is an important tool to assess the quantity and quality of current scientific production to elucidate perspectives and trends in the subject [9]. Bibliometrics is a statistical analysis tool that allows one to capture the influence of the field’s publications on the objects of study and to evaluate future trends within the research area, measuring the contributions of researchers to the investigated topic [1].

Previous reviews aimed to discuss the antimicrobial activity of postbiotics [10], the implications of paraprobiotics in intestinal dysbiosis [11], and the incorporation of probiotics and postbiotics in foods and dietary supplements [12]. However, as far as the authors of this study have noted, no current review performed a bibliometric analysis on paraprobiotics and postbiotics used by the functional food industry. Therefore, the objective of this article is, from a bibliometric analysis, to review the literature on topics related to paraprobiotics and postbiotics already studied. In this review, conceptual information, bioactivity, and future applications in the food industry are included.

## 2. Materials and Methods

### 2.1. Data Collection

The articles selected for bibliometric analysis were obtained from the Web of Science database. The search strategy consisted of thematic research on paraprobiotics and postbiotics in developing functional foods and the food industry’s path in supporting healthier diets. The Web of Science database search was performed in August 2022 using the descriptors: (paraprobiotics “OR” postbiotics) and (functional food). This search resulted in 150 results indexed in the main collection of the Web of Science. Articles published between the years 2015 and 2022 in English were considered. As a result of this filter, 7 records were excluded. A total of 143 documents were manually analyzed in terms of title, abstract, and keywords to verify the relationship and importance of the articles to the topic. From this evaluation, 132 articles were selected for bibliometric analysis.

### 2.2. Bibliometric Analysis

All data obtained from Web of Science were exported in BibTex format were analyzed using the R *Bibliometrix* package [13] and the VOSviewer version 1.6.18 tool (https://www.vosviewer.com, accessed on 8 August 2022). The R *Bibliometrix* package was used for descriptive statistical analysis and bibliometric indicators, including citation analysis, annual publication growth, authorship productivity, dominance, collaboration index, and country productivity, based on the retrieved data [14]. The VOSviewer tool is one of the most used tools in bibliometric analysis to explore the illustration networks of co-authorship, co-occurrence of keywords, citation, bibliographic coupling, and co-citation [15]. VOSviewer is a software that uses the binary counting method to extract information for each term under analysis, considering only the presence or absence of a term, failing to consider the number of occurrences of a term in the document [16]. In this review, VOSviewer was used to analyze co-authorship, citation, and co-occurrence of data collected from the documents exported from the Web of Science database.

## 3. Results

### 3.1. Description of Scientific Production and Bibliometric Analysis

Documents indexed in the Web of Science were collected from 2015, when the first reports on the subject were observed, until 2022. A detailed description of the information collected is summarized in Table 1. In 2019 there was exponential growth in the scientific literature related to paraprobiotics, postbiotics, and/or secondary metabolites formed by fermentation by probiotic lactic acid bacteria, reaching a maximum peak in 2021, with 46 articles indexed in the Web of Science database. The concept of paraprobiotics and postbiotics is recent, as is the scientific research that emerges along with the population’s demand for functional foods. From there, several areas of knowledge began to study paraprobiotics and postbiotics to understand the characteristics of possible applications.

The most relevant research area in the selected theme mainly concerns Food Technology and Science, accounting for 39.4% of the chosen publications. The second most relevant area concerns Nutritional Dietetics (17.4%), followed by Microbiology (16.7%), Microbiology Applied to Biotechnology (11.4%), and Chemistry (6.8%). These results show that the study of paraprobiotics and postbiotics has a multidisciplinary approach. Therefore, scientific progress regarding the technological approach to processing, bioactivity, and food safety is fundamental due to the need for changes in the way of developing and producing functional foods.

#### 3.1.1. Correlation between a Year of Publication, Authors’ Countries, Research Area, and Affiliation

**Figure 2:**
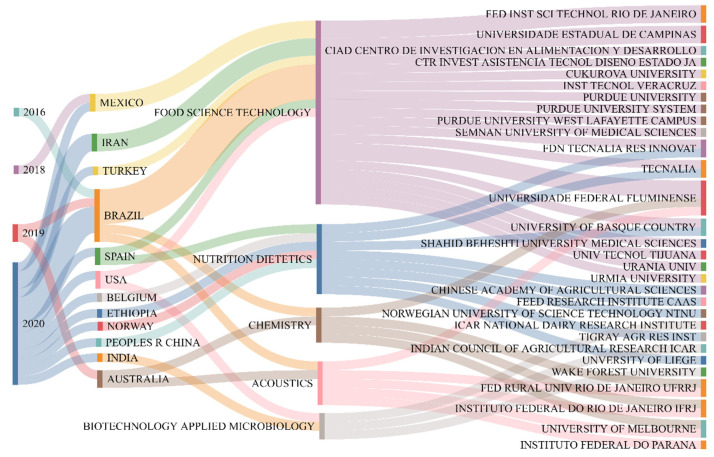
Sankey diagram of correction between a year of publication (1st column), country of authors (2nd column), area of research (3rd column), and affiliation of authors (4th column) of the 10 (ten) most cited documents. The Sankey diagram was constructed using the SankeyMATIC software (https://www.sankeymatic.com, accessed on 18 August 2022).

The bibliometric analysis that identifies countries/regions, affiliations, and areas of publications of authors and co-authors allows performance analysis of the current production. Furthermore, with advances in communication technology, people from many nations and continents can now collaborate on research projects more easily, which allows for the cross-border transfer of knowledge and technologies [17]. The correlation between the year of publication, the authors’ countries, research area, and the authors’ affiliation with the most cited documents was performed using the Sankey diagram (Figure 2). Brazil appears in the Sankey diagram as the country with the most cited scientific productions in terms of postbiotics and paraprobiotics, whose publications took place in 2016 and 2020, mainly in food science technology, chemistry, and science of sound phenomena. Brazil, Mexico, Iran, Spain, and Turkey are the leading nations with the most relevant publications in food science technology. In this area of research, the significant influence of Brazilian institutions, such as the Universidade Federal Fluminense, Instituto Federal de Educação, Ciência e Tecnologia do Rio de Janeiro, and Universidade Estadual de Campinas are also noted. Other institutions, such as the Technological Research and Development Center—Tecnalia and the Fundación Tecnalia Research and Innovation, also stand out.

Regarding dietary nutrition, the most cited documents date from 2020, with participation from countries such as Spain, Belgium, Ethiopia, Norway, and the People’s Republic of China. The collaboration of common institutions in the areas of technology of food science and chemistry is observed in this area. In addition, there are studies focused on the chemical components of functional foods and how this interaction between postbiotics, paraprobiotics, and human health occurs (e.g., Gu et al. [18]; Sawada et al. [19]; Sorensen et al. [20]).

**Figure 3:**
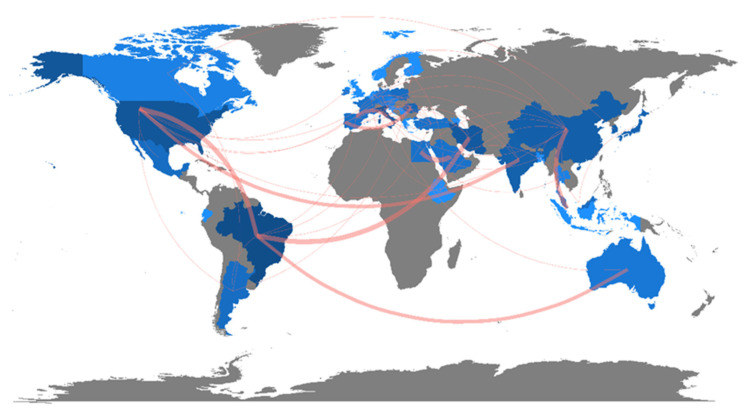
Collaboration network between authors’ countries on topics related to postbiotics and paraprobiotics.

In chemistry and acoustics, the highlight is a 2019 study produced by authors from Brazil and Australia. The Sankey diagram (Figure 2) shows the relationship and collaboration between primarily Brazilian institutions, such as the already highlighted Universidade Federal Fluminense and the University of Melbourne in Australia. The most cited documents in biotechnology applied to microbiology are from America (USA) and India. In general, terms to better understand the geographic location and the main collaborations between the authors’ countries, a world map was prepared, where it is possible to observe the collaboration network between countries (Figure 3).

The Americas are at the forefront regarding publications on the subject addressed, along with parts of Europe and Asia (Figure 3). However, it is noted that less developed regions show little or no participation in the area of study. This may be related to economic factors, where it is seen that countries with large budgets for scientific research and development have more outstanding scientific production than countries with small budgets. The relationship between scientific development and investment in science is also seen by Şahin [21]. However, in this research area, another factor that may explain the greater participation of these countries may be related to the interest, acceptance, and attitude toward the consumption of functional foods, which among Americans is more readily accepted [22]. Therefore, due to consumer demand, these countries are more interested in the technological development of functional foods with postbiotics and paraprobiotics.

#### 3.1.2. Most Cited Journals

**Figure 4:**
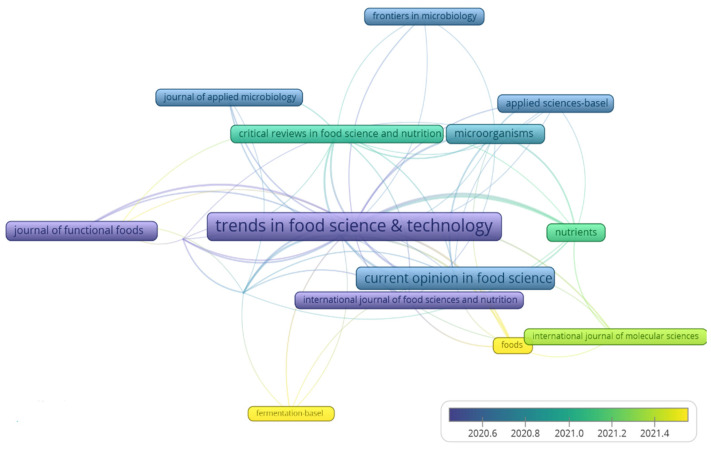
Visualization map of the similarity network of most cited journals with a minimum of four documents published between 2015 and 2022.

The analysis of document sources provides an overview of the most studied areas and how they are related to the journals that most publish and are cited by the authors. This study detected 69 journals, highlighting the journal *Food Research International* with the most significant number of documents (12), followed by *Microorganisms* (9), *Nutrients* (8), *Trends in Food Science & Technology* (5), and *Comprehensive Reviews in Food Science and Food Safety*, *Critical Reviews in Food Science and Nutrition*, *Journal of Functional Foods and LWT-Food Science and Technology*, both with four documents. Using the VOSviewer tool, it is possible to visualize the network of similarities between the journals with more than four publications between 2015 and 2022 (Figure 4). The size of the square frame and the proximity in the network map indicate the most cited journals in the evaluated documents and the similarities with the field of knowledge, respectively. The color scale lists the total citation per year. Note that these three most cited journals have an impact factor range of 4.926 to 16.002. With this, the relevance of the documents analyzed in this work is noted and provides the scientific community with important highlights of scientific production, leading to important sources to foster scientific and technological development on the application of paraprobiotics and postbiotics, especially in the area of research and development of food.

#### 3.1.3. Most Relevant Publications

The study of the relationship between paraprobiotics, postbiotics, and food is still new and, therefore, little explored. It is possible to notice this behavior through the analysis of the most cited publications, where there is a prevalence of review articles that seek to gather information, which may contribute to the future development of original research articles. Therefore, the review articles were included in this analysis of the relevance of the publications. Among the ten most cited publications, nine were reviews, and one was a research article (Table 2). The most relevant publication on the subject is a review published in the journal *Trends in Food Science & Technology* by Aguilar-Toala et al. [23]. The review highlights the recognition of the health benefits of viable bacterial cells. However, it provides an overview of new concepts such as paraprobiotics and postbiotics, drawing attention to the advantages of postbiotics, which are due to their clear chemical structure, safety in use, long shelf life since cell viability is not recognized, and content of various signaling molecules that may have anti-inflammatory, immunomodulatory, anti-obesogenic, antihypertensive, hypocholesterolemic, antiproliferative, and antioxidants [23].

**Figure 5:**
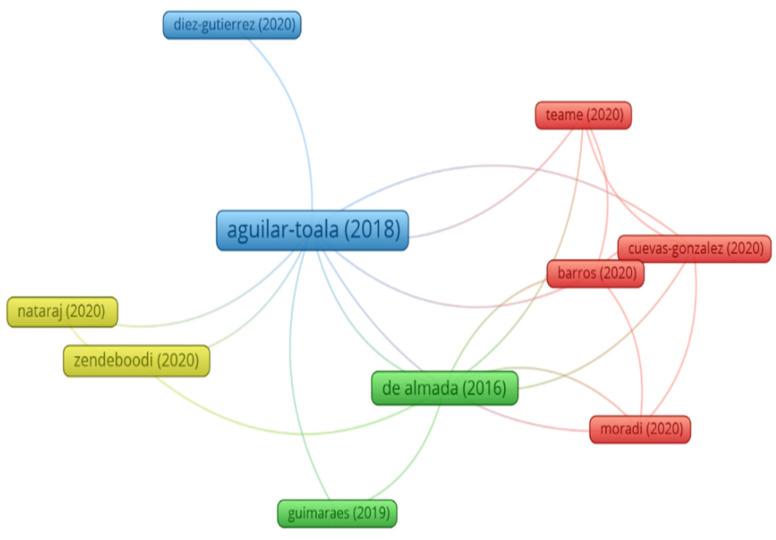
Visualization of the similarity network of the most cited documents related to paraprobiotics and postbiotics [5,6,7,8,23,24,25,26,27,28].

In Figure 5, we observe the network of collaborations between the most relevant authors researching the use and mechanism of action of paraprobiotics and postbiotics. When analyzing Figure 5, we notice the approximation of authors by the similarity of the field of study and how collaborations occur for the dissemination of scientific knowledge. The study of Aguilar-Toala et al. [23] approximates similarity to the review published by Diez-Gutiérrez et al. [28] in the *Journal of Functional Foods*, entitled “Gama-aminobutyric acid and probiotics: Multiple health benefits and their future in the global functional food and nutraceutical”. This review highlights the importance of gamma-aminobutyric acid produced by lactic acid bacteria since the imbalance of this neurotransmitter has been related to diseases of different etiologies, and the development of food products enriched with this amino acid can contribute to preventing and alleviating the symptomatology of these diseases.

The ability to modify biological responses, methods of inactivation, and perspectives on their application in food are discussed by de Almada et al. [7] in the review published in *Trends in Food Science & Technology* journal. This review highlights the use of foods as carriers of paraprobiotics as a strong field of exploration in the face of several opportunities and challenges, among them, the particular importance and selection of probiotic species to be used for the production of paraprobiotics, the use of suitable methods for inactivation and delivery, assessment of their stability and activity in food during the storage period [7]. This document, the second most cited among the documents chosen for this analysis, is close to the topic addressed by Guimarães et al. [26], which brings high-intensity ultrasound technology as a method of inactivating probiotic cultures for the development of paraprobiotic products or the improvement in the production of postbiotics. A trend in researching technologies for applying paraprobiotics and postbiotics in foods is observed according to the perceptions observed in the documents that make up this cluster.

The document entitled: “Probiotic: conceptualization from a new approach”, published in the journal *Current Opinion in Food Science* in 2020, is one of the most relevant scientific articles, which supports several studies [24]. In this document, the discussion starts with the need to conceptualize the new terms added to probiotic terminologies, such as the definitions of paraprobiotics and postbiotics. Thus, new terminology was proposed based on an efficient conceptualization of these definitions for global use, which consists of three main classes of probiotics, including the term ‘true probiotic’ to refer to viable and active probiotic cells, ‘pseudo-probiotic’ to refer to viable and inactive cells, and ‘ghost probiotic’ to refer to dead/nonviable cells, in the form of intact or ruptured cells [7]. With characteristics similar to the document produced by Zendeboodi et al. [24] and Nataraj et al. [6], published in the journal *Microbial Cell Factories*, the review entitled “Postbiotics-paraprobiotics: the new horizons in microbial biotherapy and functional foods”, where in addition to the definition presented, several implicit methodologies for extracting, purifying and identifying paraprobiotic and postbiotic compounds and their potential health benefits were comprehensively discussed.

The cluster formed by Barros et al. [25], Cuevas-González et al. [8], Moradi et al. [27], and Teame et al. [5] brings together highly cited articles since the date of publication to discuss potential applications of paraprobiotics and postbiotics in food products prepared from fermentation by lactic acid bacteria. The future perspectives for the use of paraprobiotics and postbiotics in biotechnology and the production of functional ingredients show the value of this area of study, which offers advantages over the development of probiotic products since the beneficial potential to health is not related to the viability of the bacterial cells.

#### 3.1.4. Thematic Analysis

To better understand the current interest of researchers in a specific topic, co-occurrence analysis was employed to identify the main research topics. For this, data related to keywords are collected in databases such as the Web of Science. This data can be from the author or plus keywords and reveals trends through the frequency of words used in all types of publications, including review articles [29]. The co-occurrence analysis performed in this review retrieved a total of 463 keywords from the author. The analysis was performed with a minimum occurrence of at least three keywords, resulting in 33 keywords, according to the method reported by Fasogbon and Adebo [30].

**Figure 6:**
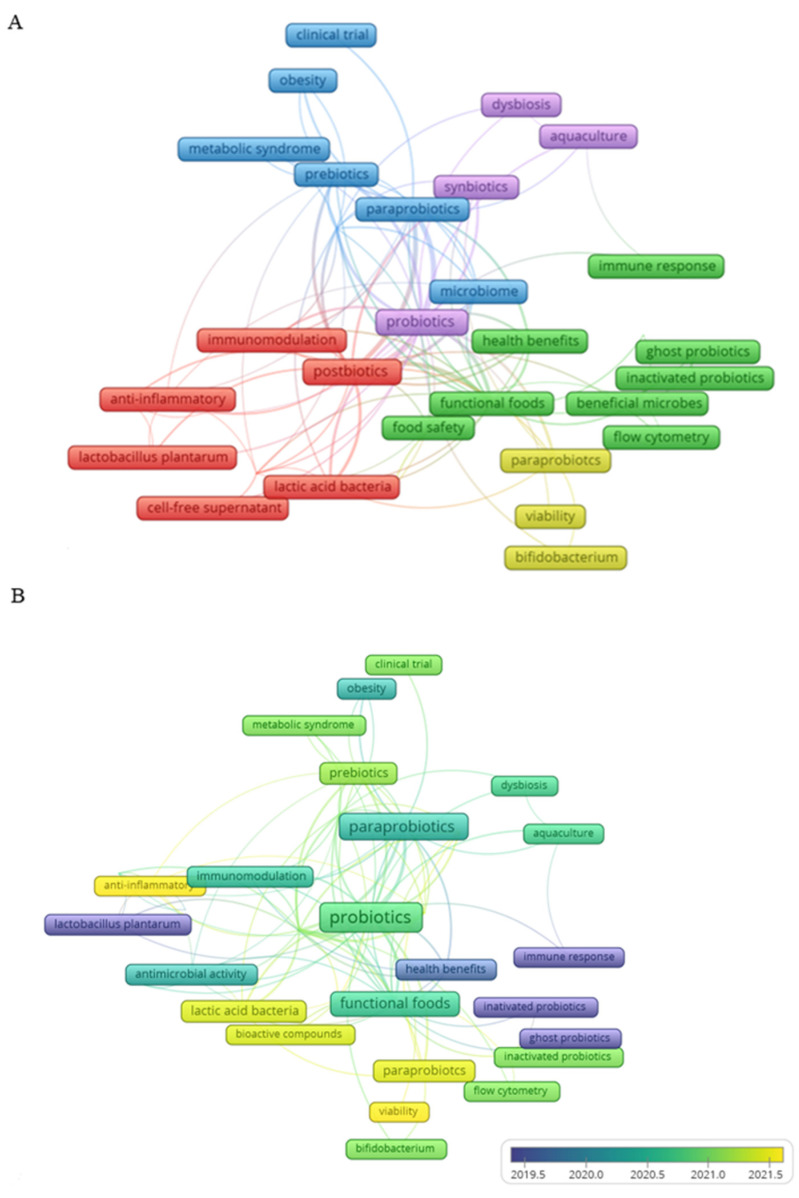
Keyword network visualization. (**A**) cluster of the main themes and (**B**) evolution of the theme by year.

In Figure 6, the similarity network of the authors’ keywords is visualized in the studies chosen for analysis. In general, it is noted that different terms are used to conceptualize the secondary metabolites produced in fermentation by probiotic bacteria and inactivated probiotic cells, as is the case with the terms “ghost probiotics” and “inactivated probiotics”. The definition and conceptualization of terms for global use in the study of these products of lactic acid bacteria beneficial to health are one of the main discussions presented by the scientific community in this area, always noting the need for a previous definition in each study. In terms of theme, in Figure 6A, we identified the formation of five clusters.

Cluster 1, in red, consists of keywords primarily targeting the beneficial health properties of postbiotics (e.g., antioxidant, immunomodulatory, and anti-inflammatory), targeting components produced by bacteria of the former *Lactobacillus* genus. The words that occur most in this cluster are postbiotics, lactic acid bacteria, and immunomodulator, respectively.

Cluster 2, in green, can be called “Functional foods”. This cluster presents food as a promise for the delivery of paraprobiotics and postbiotics, also defined by the terms “ghost probiotics” and “inactivated probiotics”. In addition to the perspective focused on the development of functional foods, another aspect is represented by the word “Food safety”. Postbiotics have significant antimicrobial properties, which allows them to be applied in combination with technologies that start to contribute to food safety practice, due to the inhibitions of the growth of food spoilage microorganisms, which contributes to the biopreservation of food and also in the control of biofilm [10].

Cluster 3, in blue, the prebiotic, paraprobiotic, and intestinal microbiota interaction is related to the benefits achieved in treating and preventing non-communicable diseases, such as obesity and obesity-associated inflammatory diseases. However, there is a high need for human/clinical trials focused on validating these health claims [6].

Cluster 4, in yellow, highlights the paraprobiotics produced by *Bifidobacterium* and the relationship with the change in bioactive compounds in food. In this cluster, there is an indication that the inactivation process to obtain paraprobiotics is a field of new research since the technology used can modify the availability of these functional components in the food or products to be developed.

Cluster 5, in purple, shows the probiotics and symbiotics targeted at aquaculture. In terms of food production, the probiotics in this cluster show an application aimed at improving fish and shrimp farming practices through therapeutic modulation strategies of the intestinal microbiota, as proposed by Vargas-Albores et al. [31]. This study presents postbiotic, paraprobiotic, and symbiotic probiotics as the most influential agents in treating dysbiosis, affecting fish health and productive performance.

In terms of the evolution of the theme over the years, in Figure 6B, a future trend is observed around food safety, identification of bioactive components with beneficial health activity, highlighting the anti-inflammatory properties, and the beneficial effects of lactic acid bacteria in general. Finally, the analysis of keywords over the years, together with the other information obtained through the bibliometric analysis described in the previous items, supported the identification of one of the most relevant topics related to postbiotics and paraprobiotics in the food industry, which is the importance of the quality and safety of food produced and consumed around the world. Insights into the future of paraprobiotics and postbiotics to contribute to food security are presented in the item below.

### 3.2. Use of Paraprobiotics and Probiotics as a Promise to Contribute to Adding Value and Food Safety

Food safety means ensuring that the food does not cause pathogenicity problems for the consumer as long as it is prepared and consumed following the recommendations for its intended use [32,33]. This issue is essential for food consumers and producers, especially as it is necessary to ensure that unsafe food does not especially harm sensitive people such as babies, young children, the elderly, and patients with low immunity [32].

The safety of probiotics has become a current concern due to reports of various side effects that have been documented for some strains, which include probiotic overgrowth in the gut, gastrointestinal symptoms, bloodstream infection, excessive D-lactase production, dysbiosis and horizontal gene transfer [34]. Given this, the possible benefits of the use of postbiotics and paraprobiotics were raised, which led to increased interest in their development and applications. Therefore, there has been increased research on postbiotics and paraprobiotics, as well as commercial and medical interest, which have become an alternative for the development of functional foods with many advantages about live probiotics (e.g., ease of storage and increased shelf life) [35]. Table 3 presents some recent applications of paraprobiotics and postbiotics with beneficial results for human health.

Zhang et al. [36] showed that heat-inactivated *Lacticaseibacillus* N1115 could alleviate antibiotic-induced damage to the gut microbiota and cognitive function, which causes anxiety and depression, by modulating the microbiota-gut-brain axis. Porfiri et al. [34] showed the immunomodulatory capacity of lactic acid bacteria strains and that this capacity can be impacted not only by different inactivation methods but also by the selected strain. The anti-adiposity effect of heat-killed *Levilactobacillus brevis* KB290 was studied by Watanabe et al. [37] using a high-fat diet-induced obesity model. It was seen that *Levilactobacillus brevis* KB290 is indicated for use as a paraprobiotic to develop functional foods that attenuate visceral fat accumulation. Almada et al. [38] showed that consumption of durum wheat pasta added with inactivated *Bifidobacterium animalis* by healthy rats reduced glucose and total cholesterol levels, revealing that durum wheat pasta is a potential vehicle for the delivery of paraprobiotics from *Bifidobacterium animalis*. Therefore, it is observed that the use of paraprobiotics is a promise for the future and of interest in several areas of science. For the food sector, using paraprobiotics is an alternative and a way to include products with safe health benefits in the market.

The interest in the use of postbiotics and paraprobiotics in the food industry goes beyond the interest in adding functional properties to foods and goes in the direction of contributing to reducing the risk of the presence of a contaminant. The former *Lactobacillus* genus is the most studied, and the paraprobiotics and postbiotics produced by bacteria of this genus consist of a wide range of cell wall polysaccharides, secreted proteins, bacteriocins, and organic acids [5]. These structural components and metabolites produced by probiotics can have diverse bioactivities, including substances with antimicrobial activity that can be used to contribute to food safety [27].

Mohammadi et al. [39] studied the application of postbiotics to develop an antimicrobial membrane by bacterial nanocellulose. They showed that postbiotics revealed antibacterial activity in all investigated strains in a concentration-dependent manner. It was also reported that when postbiotics were added to the material manufactured at a concentration of 50%, the zone of inhibition for all bacterial pathogens was greater than 20 mm, classifying these microorganisms as extremely sensitive to these postbiotics [39]. According to Chang et al. [40], the production of organic acids by *Lactiplantibacillus plantarum subsp.* during growth, such as lactic acid, reported to be predominant, promoted antimicrobial actions against salmonella growth. In addition, the ability to inhibit and prevent the oxidation process and increase antimicrobial activity has been reported, all related to the secretion of pyrrole and cyclic compounds present in postbiotics [40]. The main antimicrobial chemicals produced by *Lactobacillus* comprise ribosomally synthesized peptides (bacteriocins), metabolic by-products of various chemical natures such as hydrogen peroxide, lactic acid, and other organic acids and phenolic compounds [45]. The effective and promising use of postbiotics produced by lactic acid bacteria was also evidenced by Toushik et al. [41], where the bactericidal activity of postbiotics was reported as an alternative to the use of purified antimicrobial peptides, such as bacteriocins. Furthermore, it was seen in this article that when postbiotics derived from *Leuconostoc mesenteroides* (LAB J.27) were combined with essential oils, an excellent antimicrobial potential and efficacy as an antibiofilm was obtained.

Moradi et al. [28] highlighted in a review the most recent applications of postbiotics for ensuring food safety. The highlight is the potential use of postbiotics in food biopreservation, food packaging, and biofilm preservation and control. Biopreservation has been described as a sustainable, safe, and ecological alternative, so there has been a growing demand for studies that clarify the main application strategies and modes of action, especially for use in dairy products, meats, and products of plant origin [46]. In terms of packaging, since the deterioration of most foods occurs from the outside to the inside by the action of pathogenic microorganisms, postbiotics have great potential for application in food conservation through their use in active packaging, as a coating, forming a thin polymeric layer with postbiotics on the surface of the food, immobilized in polymers by ionic or covalent bonds, or even incorporated directly into the polymer matrix, packaging through an active film loaded with postbiotics between two outer layers, which increase postbiotics stability and control migration [27]. Another critical problem related to food safety and quality is biofilm formation, which has a more remarkable ability to survive under rigorous conditions [47]. In addition to the antimicrobial properties of lactic acid bacteria postbiotics, they can also be used as potential antibiofilm agents because of their components, including bacteriocins, organic acids, biosurfactants, and EPS, each of which can affect a particular stage of bacterial biofilm formation, due to its particular function [27].

Despite the diverse potential for the use of paraprobiotics and postbiotics, there is a need for further studies that explore the application and claims of beneficial health properties of these cellular structures and components produced by lactic acid bacteria in products, such as evaluations of the effects of adding paraprobiotic and postbiotic foods in the diet of humans and their clinical evidence, in addition to chemical, biochemical, sensory and quality and food safety characteristics. Overall, from the research addressed in this review, the prospect is for increased application of paraprobiotics and postbiotics in the food industry. However, further studies are needed to understand the best strategies for the production process.

## 4. Limitations

The bibliometric analysis presented in this study has some limitations. These limitations include the research methodology, where only the Web of Science database was used in the search. Therefore, publications not indexed in the Web of Science could not be examined and analyzed. However, 10% of the documents analyzed were manually evaluated, which shows that the method used in the study is valid and that the results obtained are accurate. Furthermore, although the VOSviewer software provides information on the link’s total strength, it is impossible to assess the number of joint publications between authors, organizations, and countries [15]. Despite the inevitable limitations of the study, we believe that the presented study can serve as a basis for future research and promote a valid discussion on the current situation of topics related to the potential application of paraprobiotics and postbiotics in food.

## 5. Conclusions

Paraprobiotics and postbiotics are new alternatives for developing quality and safe, functional foods. With the bibliometric study, it is concluded that the application of paraprobiotics and postbiotics in food is still scarce, but several review articles show the potential of beneficial health properties and production advantages compared to probiotics, which need guaranteed viability. However, there is a need for further studies on production methods, strain characteristics, and evidence of health benefits claims through in vitro and in vivo assays, as well as information on the interaction between paraprobiotics and postbiotics, and food matrices, chemical and sensory interactions and effects on the quality and safety of new products. Therefore, the application of biotic paraprobiotics in developing new functional foods is highly encouraged.

## Figures and Tables

**Table 1:** Descriptive analysis of documents collected from the Web of Science database in August 2022.

Description	Results
Main information about data	
Timespan	2015–2022
Sources	69
Documents	132
Annual growth rate (%)	66.18
Document average age	1.32
Average citations per doc	14.33
References	9626
Document contents	
Keywords plus	6637
Author’s Keywords	460
Authors	
Authors	620
Authors of single-authored docs	4
Authors collaboration	
Single-authored docs	4
Co-authors per doc	5.62
International co-authorships (%)	30.3
Document types	
Article	77
Article; book chapter	1
Article; early access	2
Review	47
Review, early access	5

**Table 2:** Main scientific articles on paraprobiotics and postbiotics.

Reference	Document Type	Main Results	Average Citation per Year	Total Citation
Aguilar-Toalá et al. [23]	Review	- Postbiotics are metabolites and/or components secreted by probiotic microorganisms. - Postbiotics can, along with probiotics, improve the host’s health. - The mechanisms by which postbiotics bioactivity are not yet fully elucidated.	54.4	272
De Almada et al. [7]	Review	- Similar to probiotics, paraprobiotics are capable of modifying biological responses in living beings. - The correlation between cellular physical properties, viability, and biological properties approaches for the development of the developing paraprobiotic products. - The development of paraprobiotics as an ingredient will serve many applications where adding probiotics is a technological challenge.	17.71	124
Zendeboodi et al. [24]	Article	- Existing terminologies about probiotics were criticized and new terminologies were proposed and described. - The mentioned types or states of probiotics and their health impacts and mechanisms of action have been discussed concisely.	36	108
Nataraj et al. [6]	Review	- Paraprobiotics and postbiotics have advantages over probiotics, such as better availability of production process for industrial scale and ease of production and storage. - There is a high need for human/clinical trials to validate health claims. - I vitro and in vivo studies of the stability of paraprobiotics and postbiotics under digestive conditions are necessary to better understand the bioactive activities of these products.	29.67	89
Barros et al. [25]	Review	- The term psychobiotics indicates probiotics with mental health benefits. - The main advantages around the application of paraprobiotics and postbiotics for dairy food production were identified. - A survey and analysis of methods for identifying and quantifying paraprobiotics and postbiotics were carried out.	20.67	62
Guimaraes et al. [26]	Review	- The effects of high-intensity ultrasound on probiotics and prebiotic dairy products have been described. - Ultrasound technology can be used to improve the functional activities of foods and for the development of new probiotic and prebiotic dairy products.	15.5	62
Cuevas-Gonzales et al. [8]	Review	- The health benefits attributed to postbiotics and paraprobiotics do not require cell viability. - Protective effect on cell lines of animal models was attributed to postbiotics and paraprobiotics. - To support claims of health benefits and effectiveness in treating disease, human trials are needed.	19.67	59
Moradi et al. [27]	Review	- Possible applications of postbiotics in food biopreservation, food packaging, and biofilm control were reviewed. - Postbiotics are considered in the reduction and biodegradation of some chemical contaminants related to food safety.	17	51
Diez-Gutierrez et al. [28]	Review	- Postbiotics such as gamma-aminobutyric acid enhance the probiotic health effect. - Gama-aminobutyric acid plays an essential role in preventing neural diseases, type 1 diabetes, cancer, immune disorders, and asthma.	16	48
Teame et al. [5]	Review	- Like functional foods, paraprobiotics and postbiótics have good potential for human or animal use as prophylactic or therapeutic agents. - Paraprobiotics and postbiotics derived from *Lactobacillus* species have beneficial functions in preserving the epithelial barrier, antitumor effect, immunomodulation, and antagonistic effects against pathogens.	15.33	46

**Table 3:** Main applications of paraprobiotics and probiotics.

Microorganisms	Application	Highlights	References
*Lacticaseibacillus* N1115	Brain dysfunction treatment	Significant relief from memory dysfunction, anxiety, and depression.	[36]
*Lacticaseibacillus casei*, *Lactobacillus acidophilus*, *Lactiplantibacillus plantarum*, and *Lacticaseibacillus paracasei*	Immunology modulation	The immunomodulatory activity can be altered according to the inactivation treatment.	[34]
*Levilactobacillus brevis* KB290	Reduction of fat accumulation	Reduction of elevated serum glucose levels and insulin resistance.	[37]
*Bifidobacterium animalis*	Durum wheat pasta	Reduction in serum glucose and cholesterol levels.	[38]
Postbiotics de FreshQ (*Lacticaseibacillus rhamnosus*, *Lacticaseibacillus paracasei subsp.*)	Packaging material	Antimicrobial activity.	[39]
*Lactiplantibacillus plantarum*	Antioxidant agent	The composition and functional characteristics of postbiotics are influenced by the strain and composition of the culture medium.	[40]
*Leuconostoc mesenteroides* J27	Antibiofilm	Postbiotics and essential oils can efficiently inhibit biofilm formation in seafood.	[41]
*Latilactobacillus curvatus* B67	Antimicrobial and antibiofilm	Inhibition of pathogenic biofilm formation, which indicates potential use as an alternative bioprotective agent in the meat processing industry.	[42]
*E. coli Nissle 1917*	Functional Yogurt	Antimicrobial, antitumor, and antioxidant activities.	[43]
*Pediococcus. Acidilactici*; *Latilactobacillus sakei/Staphylococcus xylosus*	chicken thighs	It reduced pathogens in chicken thighs, which could contribute to extending the shelf life of poultry meat and meat products.	[44]

## Data Availability

Not applicable.

## References

[1] Pazzini I.A.E., de Melo A.M., Ribani R.H. (2021). Bioactive Potential, Health Benefits and Application Trends of *Syzygium malaccense* (Malay Apple): A Bibliometric Review. Trends Food Sci. Technol..

[2] Pimentel T.C., Brandão L.R., de Oliveira M.P., da Costa W.K.A., Magnani M. (2021). Health Benefits and Technological Effects of *Lacticaseibacillus casei*-01: An Overview of the Scientific Literature. Trends Food Sci. Technol..

[3] Meng F., Zhao M., Lu Z. (2022). The LuxS/AI-2 System Regulates the Probiotic Activities of Lactic Acid Bacteria. Trends Food Sci. Technol..

[4] Hill C., Guarner F., Reid G., Gibson G.R., Merenstein D.J., Pot B., Morelli L., Canani R.B., Flint H.J., Salminen S. (2014). The International Scientific Association for Probiotics and Prebiotics Consensus Statement on the Scope and Appropriate Use of the Term Probiotic. Nat. Rev. Gastroenterol. Hepatol..

[5] Teame T., Wang A., Xie M., Zhang Z., Yang Y., Ding Q., Gao C., Olsen R.E., Ran C., Zhou Z. (2020). Paraprobiotics and Postbiotics of Probiotic *Lactobacilli*, Their Positive on the Host and Action Mechanisms: A Review. Front. Nutr..

[6] Nataraj B.H., Ali S.A., Behare P.V., Yadav H. (2020). Postbiotics-Parabiotics: The New Horizons in Microbial Biotherapy and functional Foods. Microb. Cell Fact..

[7] De Almada C.N., Almada C.N., Martinez R.C.R., Sant’Ana A.S. (2016). Paraprobiotics: Evidences on Their Ability to Modify Biological Responses, Inactivation Methods and Perspectives on Their Application in Foods. Trends Food Sci. Technol..

[8] Cuevas-Gonzalez P.F., Liceaga A.M., Aguilar-Toala J.E. (2020). Postbiotics and Paraprobiotics: From Concepts to Applications. Food Res. Int..

[9] Wang J., Maniruzzaman M.A. (2022). Global Bibliometric and Visualized Analysis of Bacteria-Mediated Cancer Therapy. Drug Discov. Today.

[10] Rad A.H., Hosseini S., Pourjafar H. (2022). Postbiotics as Dynamic Biological Molecules for Antimicrobial Activity: A Mini-Review. Biointerface Res. Appl. Chem..

[11] Rahman Z., Dandekar M.P. (2022). Implication of Paraprobiotics in Age-Associated Gut Dysbiosis and Neurodegenerative Diseases. NeuroMol. Med..

[12] Puntillo M., Segli F., Champagne C.P., Raymond Y., Vinderola G. (2022). Functional Microbes and Their Incorporation into Foods and Food: Probiotics and Postbiotics. Annu. Rev. Food Sci. Technol..

[13] Aria M., Cuccurullo C. (2017). Bibliometrix: An R-Tool for Comprehensive Science Mapping Analysis. J. Informetr..

[14] Anglada-Tort M., Sanfilippo K.R.M. (2019). Visualizing Music Psychology: A Bibliometric Analysis of *Psychology of Music*, *Music Perception*, and *Musicae Scientiae* from 1973 to 2017. Music. Sci..

[15] Silva R., Rocha R.S., Ramos G.L.P.A., Xavier-Santos D., Pimentel T.C., Lorenzo J.M., Henrique Campelo P., Cristina Silva M., Esmerino E.A., Freitas M.Q. (2022). What Are the Challenges for Ohmic Heating in the Food Industry? Insights of a Bibliometric Analysis. Food Res. Int..

[16] Colares G.S., Dell’Osbel N., Wiesel P.G., Oliveira G.A., Lemos P.H.Z., da Silva F.P., Lutterbeck C.A., Kist L.T., Machado Ê.L. (2020). Floating Treatment Wetlands: A Review and Bibliometric Analysis. Sci. Total Environ..

[17] Nordin A.H., Ngadi N., Ilyas A.R., Nabgan W., Norfarhana A.S. (2022). Starch-Based Plastics: A Bibliometric Analysis. Mater. Today Proc..

[18] Gu Z., Meng S., Wang Y., Lyu B., Li P., Shang N. (2022). A Novel Bioactive Postbiotics: From Microbiota-Derived Extracellular to Health Promoting. Crit. Rev. Food Sci. Nutr..

[19] Sawada D., Sugawara T., Hirota T., Nakamura Y. (2022). Effects of *Lactobacillus gasseri* CP2305 on Mild Menopausal Symptoms In-Aged Women. Nutrients.

[20] Sorensen H.M., Rochfort K.D., Maye S., MacLeod G., Brabazon D., Loscher C., Freeland B. (2022). Exopolysaccharides of Lactic Acid Bacteria: Production, Purification and Health Benefits towards Functional Food. Nutrients.

[21] Şahin E. (2022). A Bibliometric Overview of the International Journal of Gastronomy and Food Science: To Where Is Gastronomy Research Evolving?. Int. J. Gastron. Food Sci..

[22] Tur J.A., Bibiloni M.M. (2016). Functional Foods. Encyclopedia of Food and Health.

[23] Aguilar-Toalá J.E., Garcia-Varela R., Garcia H.S., Mata-Haro V., González-Córdova A.F., Vallejo-Cordoba B., Hernández-Mendoza A. (2018). Postbiotics: An Evolving Term within the Functional Foods Field. Trends Food Sci. Technol..

[24] Zendeboodi F., Khorshidian N., Mortazavian A.M., da Cruz A.G. (2020). Probiotic: Conceptualization from a New Approach. Curr. Opin. Food Sci..

[25] Barros C.P., Guimaraes J.T., Esmerino E.A., Duarte M.C.K.H., Silva M.C., Silva R., Ferreira B.M., Sant’Ana A.S., Freitas Monica Q., Cruz A.G. (2020). Paraprobiotics and Postbiotics: Concepts and Potential Applications in dairy Products. Curr. Opin. Food Sci..

[26] Guimarães J.T., Balthazar C.F., Scudino H., Pimentel T.C., Esmerino E.A., Ashokkumar M., Freitas M.Q., Cruz A.G. (2019). High-Intensity Ultrasound: A Novel Technology for the Development of probiotic and Prebiotic Dairy Products. Ultrason. Sonochem..

[27] Moradi M., Kousheh S.A., Almasi H., Alizadeh A., Guimaraes J.T., Yilmaz N., Lotfi A. (2020). Postbiotics Produced by Lactic Acid Bacteria: The next Frontier in Food. Compr. Rev. Food Sci. Food Saf..

[28] Diez-Gutierrez L., San Vicente L., Barron Luis Javier R., del Carmen Villaran M., Chavarri M. (2020). Gamma-Aminobutyric Acid and Probiotics: Multiple Health Benefits and their Future in the Global Functional Food and Nutraceuticals Market. J. Funct. Foods.

[29] de Souza W.F.C., Almeida F.L.C., de Castro R.J.S., Sato H.H. (2022). Isomaltulose: From Origin to Application and Its Beneficial Properties—A Bibliometric Approach. Food Res. Int..

[30] Fasogbon B.M., Adebo O.A. (2022). A Bibliometric Analysis of 3D Food Printing Research: A Global and African Perspective. Future Foods.

[31] Vargas-Albores F., Martinez-Cordova L.R., Hernandez-Mendoza A., Cicala F., Lago-Leston A., Martinez-Porchas M. (2021). Therapeutic Modulation of Fish Gut Microbiota, a Feasible Strategy For?. Aquaculture.

[32] Rad A.H., Aghebati-Maleki L., Kafil Hossein Samadi and Abbasi A. (2021). Molecular Mechanisms of Postbiotics in Colorectal Cancer Prevention And. Crit. Rev. Food Sci. Nutr..

[33] De Freitas R.S.G., da Cunha D.T., Stedefeldt E. (2019). Food Safety Knowledge as Gateway to Cognitive Illusions of Food Handlers and the Different Degrees of Risk Perception. Food Res. Int..

[34] Porfiri L., Burtscher J., Kangethe R.T., Verhovsek D., Cattoli G., Domig K.J., Wijewardana V. (2022). Irradiated Non-Replicative Lactic Acid Bacteria Preserve Metabolic While Exhibiting Diverse Immune Modulation. Front. Vet. Sci..

[35] Mi X.J., Tran T.H.M., Park H.R., Xu X.Y., Subramaniyam S., Choi H.S., Kim J., Koh S.C., Kim Y.J. (2022). Immune-Enhancing Effects of Postbiotic Produced by *Bacillus velezensis*-2 Isolated from Korea Foods. Food Res. Int..

[36] Zhang Y., Liang H., Wang Y., Cheng R., Pu F., Yang Y., Li J., Wu S., Shen X., He F. (2022). Heat-Inactivated *Lacticaseibacillus paracasei* N1115 Alleviates the damage Due to Brain Function Caused by Long-Term Antibiotic Cocktail in Mice. BMC Neurosci..

[37] Watanabe J., Hashimoto N., Yin T., Sandagdorj B., Arakawa C., Inoue T., Suzuki S. (2021). Heat-killed *Lactobacillus brevis* KB290 Attenuates Visceral Fat Accumulation Induced by High-fat Diet in Mice. J. Appl. Microbiol..

[38] Almada C.N., Almada-Érix C.N., Costa W.K., Graça J.S., Cabral L., Noronha M.F., Gonçalves A.E.S., Santos A.D., Lollo P.C., Magnani M. (2021). Wheat-Durum Pasta Added of Inactivated *Bifidobacterium animalis* Glucose and Total Cholesterol Levels and Modulates Gut in Healthy Rats. Int. J. Food Sci. Nutr..

[39] Mohammadi R., Moradi M., Tajik H., Molaei R. (2022). Potential Application of Postbiotics Metabolites from Bioprotective Culture to Fabricate Bacterial Nanocellulose Based Antimicrobial Packaging Material. Int. J. Biol. Macromol..

[40] Chang H.M., Foo H.L., Loh T.C., Lim E.T.C., Abdul Mutalib N.E. (2021). Comparative Studies of Inhibitory and Antioxidant Activities, and Organic Acids Compositions of Postbiotics Produced by Probiotic *Lactiplantibacillus plantarum* Strains Isolated from Malaysian Foods. Front. Vet. Sci..

[41] Toushik S.H., Park J.H., Kim K., Ashrafudoulla M., Ulrich M.S.I., Mizan M.F.R., Roy P.K., Shim W.B., Kim Y.M., Park S.H. (2022). Antibiofilm Efficacy of *Leuconostoc mesenteroides* J.27-Derived Postbiotic and Food-Grade Essential Oils against *Vibrio parahaemolyticus*, *Pseudomonas aeruginosa*, and *Escherichia coli* Alone and in Combination, and Their Application as a Green Preservative in the Seafood Industry. Food Res. Int..

[42] Toushik S.H., Kim K., Park S.H., Park J.H., Ashrafudoulla M., Ulrich M.S.I., Mizan M.F.R., Hossain M.I., Shim W.B., Kang I. (2023). Prophylactic Efficacy of *Lactobacillus curvatus* B67-Derived Postbiotic and Quercetin, Separately and Combined, against *Listeria monocytogenes* and *Salmonella enterica* ser. Typhimurium on Processed Meat Sausage. Meat Sci..

[43] Darwish M.S., Qiu L., Taher M.A., Zaki A.A., Abou-Zeid N.A., Dawood D.H., Shalabi O.M.A.K., Khojah E., Elawady A.A. (2022). Health Benefits of Postbiotics Produced by E. Coli Nissle 1917 in Functional Yogurt Enriched with Cape Gooseberry (*Physalis peruviana* L.). Fermentation.

[44] İncili G.K., Karatepe P., Akgöl M., Güngören A., Koluman A., İlhak O.İ., Kanmaz H., Kaya B., Hayaloğlu A.A. (2022). Characterization of Lactic Acid Bacteria Postbiotics, Evaluation in-Vitro Antibacterial Effect, Microbial and Chemical Quality on Chicken Drumsticks. Food Microbiol..

[45] Rocchetti M.T., Russo P., Capozzi V., Drider D., Spano G., Fiocco D. (2021). Bioprospecting Antimicrobials from *Lactiplantibacillus plantarum*: Key Underlying Its Probiotic Action. Int. J. Mol. Sci..

[46] Hernández A., Rodríguez A., Córdoba M.G., Martín A., Ruiz-Moyano S. (2022). Fungal Control in Foods through Biopreservation. Curr. Opin. Food Sci..

[47] Zhu T., Yang C., Bao X., Chen F., Guo X. (2022). Strategies for Controlling Biofilm Formation in Food Industry. Grain Oil Sci. Technol..

